# Comparison of expandable and titanium cages in anterior cervical corpectomy for OPLL with osteoporosis: A retrospective matched study

**DOI:** 10.1097/MD.0000000000044227

**Published:** 2025-09-12

**Authors:** Yaqi Li, Kexi Yang, Zhancheng Liang, Jialing Liang, Hanhui Liu

**Affiliations:** a Department of Spine Surgery, Foshan Fosun Chancheng Hospital, Foshan, China.

**Keywords:** cervical ossification of the posterior longitudinal ligament (OPLL), expandable vertebral body cage, osteoporosis, titanium cage fusion

## Abstract

Anterior cervical corpectomy and fusion (ACCF) is commonly used to treat cervical ossification of the posterior longitudinal ligament (OPLL). In patients with concurrent osteoporosis, titanium cage fusion (TCF) may lead to increased implant subsidence and poorer early outcomes. Novel expandable artificial vertebral body fusion (NEAVBF) systems have been developed to address these challenges by offering improved intraoperative adjustability and biomechanical support. To compare clinical and radiographic outcomes of NEAVBF and TCF in osteoporotic patients with cervical OPLL undergoing single-level ACCF. Retrospective cohort study with 1:1 propensity score matching. Eighty-two patients with cervical OPLL and osteoporosis (41 in each group) who underwent single-level ACCF and completed at least 2 years of follow-up. Radiographic outcomes included C2 to C7 Cobb angle, implant subsidence, and fusion rate. Clinical outcomes included Visual Analogue Scale, Neck Disability Index, Japanese Orthopaedic Association (JOA) score, and JOA recovery rate at preoperative, 6-month, and 2-year follow-up. Outcomes were compared between groups at preoperative, 6-month, and 2-year time points. Both groups showed significant improvements in pain and neurological function. At 6 months, NEAVBF resulted in lower Visual Analogue Scale (*P* < .001), higher JOA scores (*P* = .013), and greater recovery rate (*P* < .001), while Neck Disability Index scores were similar (*P* = .065). Cervical lordosis improved more in the NEAVBF group at 6 months (*P* = .038) and 2 years (*P* = .004). Implant subsidence was less common in the NEAVBF group at 6 months (12.2% vs 31.7%, *P* = .060) and 2 years (26.8% vs 53.7%, *P* = .024). Although 6-month fusion rates were higher in the NEAVBF group (70.7% vs 51.2%, *P* = .112), both groups reached similar fusion at 2 years (97.6% vs 92.7%, *P* = .616). Both NEAVBF and TCF provided favorable long-term outcomes. However, NEAVBF demonstrated superior early cervical alignment, neurological recovery, and reduced implant subsidence, supporting its value in osteoporotic patients with cervical OPLL.

## 1. Introduction

Ossification of the posterior longitudinal ligament (OPLL) is a pathological condition characterized by ectopic calcification of the posterior longitudinal ligament, most commonly affecting the cervical spine.^[1]^ This progressive ossification leads to narrowing of the spinal canal and subsequent compression of the spinal cord and nerve roots.^[2]^ Clinically, cervical OPLL presents with varying degrees of myelopathy, including gait disturbances, upper extremity weakness, sensory deficits, and impaired fine motor coordination.^[3]^ As the disease progresses, neurological deterioration becomes more likely, necessitating timely surgical intervention to prevent irreversible spinal cord injury.

Anterior cervical corpectomy and fusion (ACCF) is a well-established surgical procedure for multilevel OPLL, as it enables direct decompression of the spinal cord and removal of the ossified ligament.^[4]^ However, managing OPLL in patients with coexisting osteoporosis remains challenging. Decreased bone mineral density (BMD) compromises vertebral endplate integrity, increasing the risk of implant subsidence, fusion failure, and postoperative complications.^[5,6]^ In this high-risk population, achieving and maintaining spinal stability after ACCF requires careful selection of the implant strategy and consideration of biomechanical demands.^[7]^

Titanium mesh cages are commonly used in ACCF to reconstruct the anterior column following corpectomy.^[8]^ While effective in many patients, their rigid structure and limited endplate conformity may result in uneven load distribution and implant subsidence, particularly in osteoporotic bone.^[9]^ Subsidence can adversely affect cervical alignment, compromise fusion, and deteriorate long-term clinical outcomes.^[10]^

Expandable vertebral body systems have emerged as an alternative with potential advantages. These implants allow intraoperative adjustment of height and angle, improving endplate contact and load distribution.^[11]^ Such features may enhance initial biomechanical stability and reduce the risk of subsidence in osteoporotic patients.^[12]^ However, clinical evidence comparing expandable cages with titanium cages remains limited, especially in high-risk populations such as those with both OPLL and osteoporosis.

To address this gap, we conducted a retrospective propensity score-matched study to compare the clinical and radiographic outcomes of novel expandable artificial vertebral body fusion (NEAVBF) and titanium cage fusion (TCF) in patients with cervical OPLL and osteoporosis undergoing ACCF. This study focused on key postoperative parameters, including cervical alignment, implant subsidence, neurological recovery, and fusion rates. To our knowledge, this is the first study to directly compare these 2 implant strategies in this high-risk population using matched cohorts. We hypothesized that NEAVBF would provide superior early spinal stability, reduce the incidence of implant subsidence, and improve neurological outcomes compared with conventional titanium cages.

## 2. Materials and methods

### 2.1. Study design and population

This retrospective observational study was approved by the Ethics Committee of Foshan Fosun Chancheng Hospital (approval number: FXLL20241181). Given the retrospective design and anonymized data collection, the requirement for written informed consent was waived for the review of clinical records and imaging data.

Patients diagnosed with cervical OPLL combined with osteoporosis who underwent single-level ACCF between August 2022 and August 2024 were retrospectively reviewed. Osteoporosis was diagnosed based on dual-energy X-ray absorptiometry with a *T*-score ≤ –2.5 at the lumbar spine or femoral neck, consistent with World Health Organization criteria.^[13]^ Preoperative evaluation included detailed clinical assessment, neurological examination, and imaging studies with lateral cervical spine X-rays, computed tomography (CT) scans to assess the extent and morphology of OPLL, and magnetic resonance imaging to evaluate spinal cord compression and signal changes. This multimodal imaging approach confirmed the diagnosis and provided baseline radiographic parameters such as the spinal canal occupying ratio, which was included in the baseline comparison (Table 1).

**Table 1:** Baseline characteristics of the patients in the NEAVBF and TCF groups included in the unmatched and matched cohorts.

	Unmatched cohorts			Matched cohorts		
	NEAVBF (n = 41)	TCF (n = 92)	*P*-value	NEAVBF (n = 41)	TCF (n = 41)	*P*-value
Patient demographics						
Age	62.83 ± 5.72	64.18 ± 6.44	0.251	62.83 ± 5.72	62.70 ± 6.45	.921
Sex (M:F)	24:17	44:48	0.340	24:17	28:13	.492
Hypertension	22 (53.7%)	40 (43.5%)	0.369	22 (53.7%)	22 (53.7%)	1.000
Diabetes	4 (9.8%)	17 (18.5%)	0.309	4 (9.8%)	2 (4.9%)	.672
Smoking	17 (41.5%)	20 (21.7%)	0.033*	17 (41.5%)	15 (36.6%)	.821
Drinking	12 (29.3%)	21 (22.8%)	0.564	12 (29.3%)	9 (22.0%)	.613
Preoperative radiographic measurements						
Bone density (T-score)	-2.86 ± 0.27	-2.72 ± 0.26	0.006*	-2.86 ± 0.27	-2.82 ± 0.27	.522
SCOR (%)	39.95 ± 12.73	40.37 ± 11.95	0.855	39.95 ± 12.73	39.64 ± 12.55	.911
C2–C7 Cobb angle (°)	8.14 ± 2.08	7.58 ± 2.11	0.156	8.14 ± 2.08	8.48 ± 2.22	.480
PROM						
VAS	5.27 ± 1.05	5.61 ± 1.14	0.105	5.27 ± 1.05	5.46 ± 1.07	.408
NDI	31.85 ± 8.77	31.20 ± 8.97	0.695	31.85 ± 8.77	31.29 ± 8.29	.767
JOA	10.85 ± 0.34	10.96 ± 0.32	0.077	10.85 ± 0.34	10.97 ± 0.32	.102

JOA = Japanese Orthopedic Association, NDI = Neck Disability Index, NEAVBF = novel expandable artificial vertebral body fusion, PROM = patient-reported outcome measures, SCOR = spinal canal occupying ratio, TCF = titanium cage fusion, VAS = Visual Analogue Scale.

* Statistically significant difference between the 2 groups.

Anterior column reconstruction was performed using either a novel expandable artificial vertebral body system (T2 Stratosphere Expandable Corpectomy System, Medtronic Sofamor Danek, USA) or a titanium cage packed with autologous bone graft.

**Figure 1.**
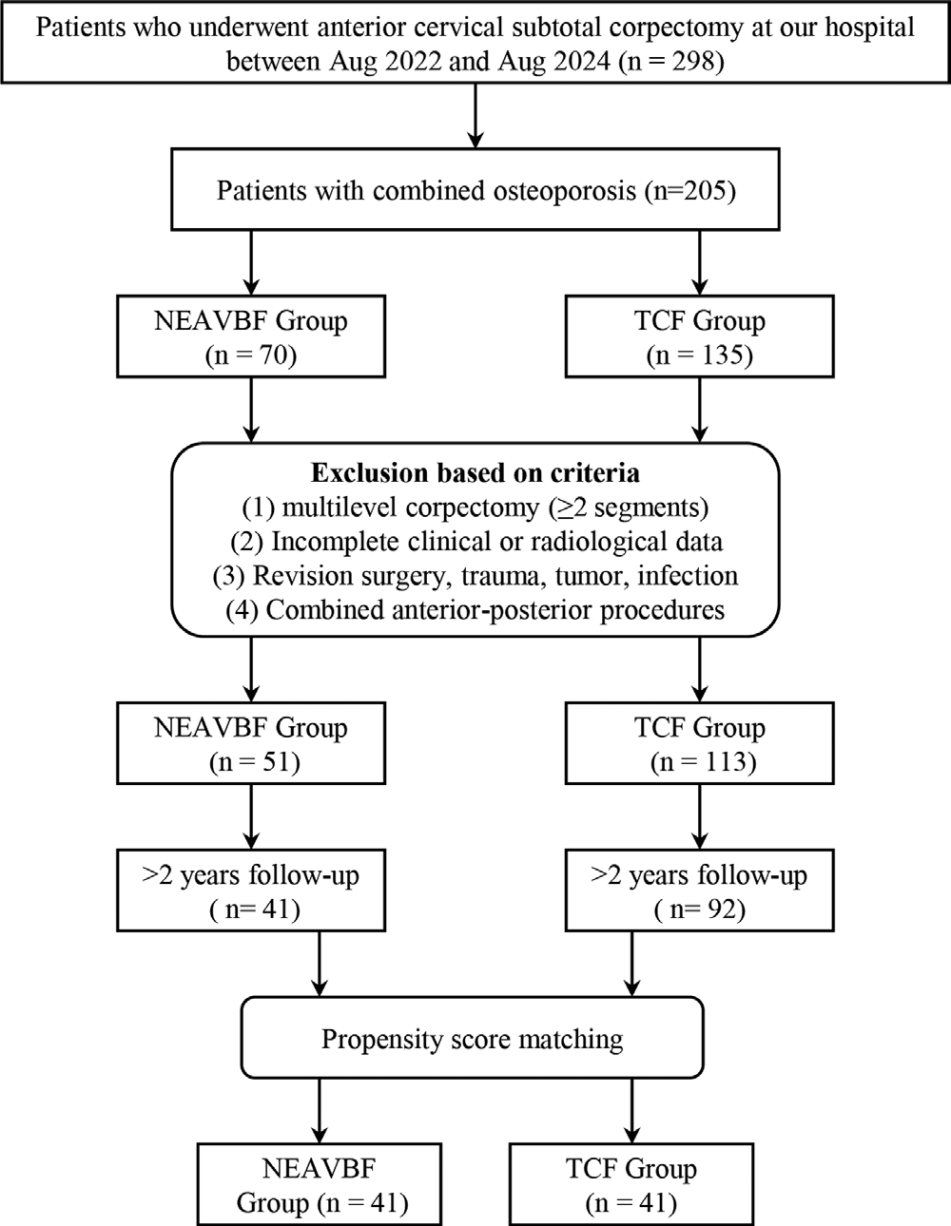
Patient selection process. NEAVBF = novel expandable artificial vertebral body fusion, TCF = titanium cage fusion.

A total of 133 patients met the inclusion criteria and completed a minimum of 24 months of follow-up. Exclusion criteria were as follows: multilevel corpectomy involving 2 or more vertebral segments; incomplete clinical or radiological follow-up data; history of cervical trauma, infection, tumor, or revision surgery; and combined anterior–posterior cervical procedures (Fig. 1).

Eligible patients were categorized into 2 groups according to the anterior column reconstruction device they received: the NEAVBF group (n = 41) and the TCF group (n = 41). All surgeries were performed by the same surgical team. Postoperative follow-up was scheduled at 6 and 24 months and included standardized clinical assessments (Japanese Orthopaedic Association [JOA], Visual Analog Scale [VAS], and Neck Disability Index [NDI] scores) and imaging evaluations. Lateral X-rays were routinely performed at both follow-up time points to measure C2 to C7 Cobb angle and detect implant subsidence. CT scans were obtained selectively when fusion status could not be clearly determined on plain radiographs.

### 2.2. Surgical techniques

All patients underwent surgery under general anesthesia using a standard right-sided anterior cervical approach. After confirming the target level via intraoperative fluoroscopy, a transverse skin incision was made, and the prevertebral space was exposed through blunt dissection.

Subtotal corpectomy of the involved vertebral body was performed using a high-speed drill and Kerrison rongeurs. The adjacent intervertebral discs were removed, and decompression of the spinal cord was completed by carefully resecting the posterior vertebral wall and the OPLL under microscopic visualization. The surrounding vertebral endplates were preserved to maintain structural integrity and enhance the likelihood of successful fusion.

**Figure 2.**
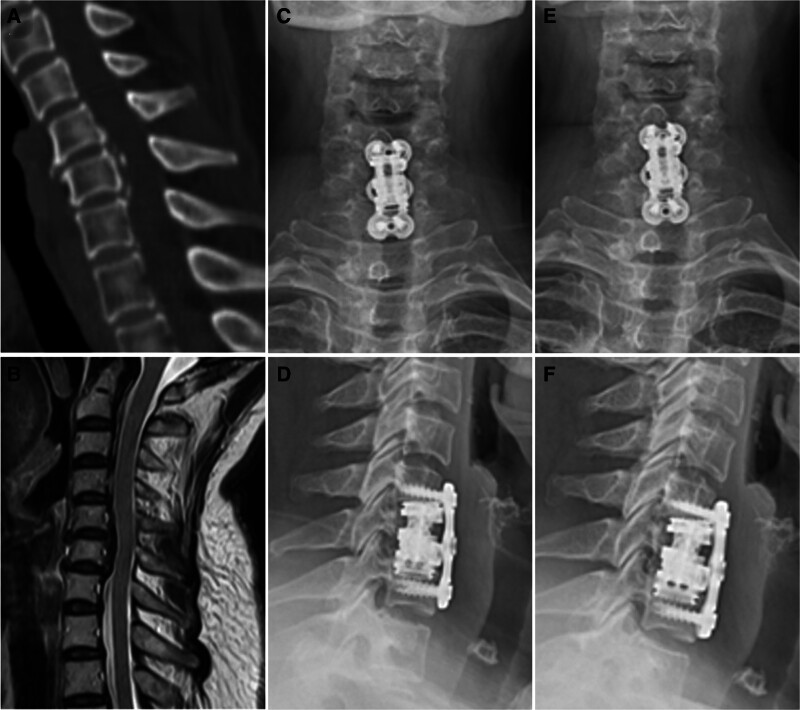
Representative radiographic images of a patient in the NEAVBF group who underwent single-level ACCF for cervical OPLL with osteoporosis. (A) Preoperative sagittal computed tomography (CT) image showing segmental OPLL with canal narrowing. (B) Preoperative sagittal T2-weighted magnetic resonance imaging (MRI) demonstrating spinal cord compression at the involved level. (C, D) Anteroposterior and lateral radiographs at 6 months postoperatively showing satisfactory implant position, restoration of cervical alignment, and no evidence of subsidence. (E, F) Radiographs at 2 years postoperatively showing maintained alignment, stable implant fixation, and solid fusion without further subsidence. ACCF = anterior cervical corpectomy and fusion, NEAVBF = novel expandable artificial vertebral body fusion, OPLL = ossification of the posterior longitudinal ligament.

In the NEAVBF group, reconstruction of the anterior column was achieved using a novel expandable artificial vertebral body (T2 Stratosphere Expandable Corpectomy System, Medtronic, USA). The device was inserted in a collapsed state and gradually expanded in situ to restore disc height and cervical lordosis. It was then locked at the desired height, and anterior fixation was applied using a cervical plate and screws (Fig. 2).

**Figure 3.**
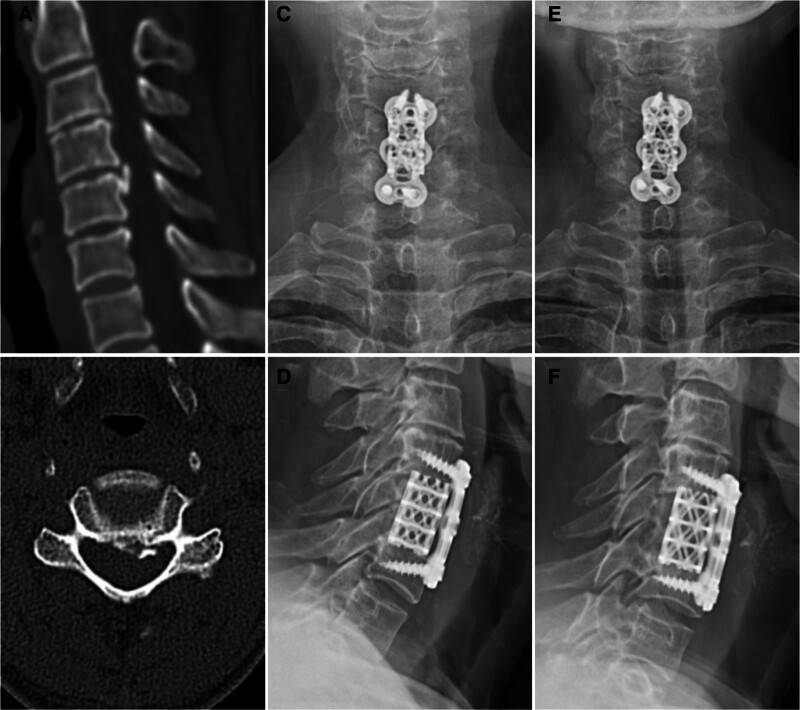
Representative radiographic images of a patient in the TCF group who underwent single-level ACCF for cervical OPLL. (A, B) Preoperative sagittal and axial CT images showing segmental ossification with canal narrowing. (C, D) Anteroposterior and lateral radiographs at 6 months postoperatively demonstrating proper implant position. (E, F) Radiographs at 2 years postoperatively showing implant stability and bony fusion with mild subsidence. ACCF = anterior cervical corpectomy and fusion, CT = computed tomography, OPLL = ossification of the posterior longitudinal ligament, TCF = titanium cage fusion.

In the TCF group, an appropriately sized titanium mesh cage was selected and filled with autologous bone harvested from the resected vertebra. The cage was inserted into the corpectomy gap, followed by the application of an anterior cervical plate to stabilize the construct (Fig. 3).

Intraoperative C-arm fluoroscopy was used throughout to verify implant position, alignment, and screw fixation. Hemostasis was achieved, and a closed-suction drain was placed before wound closure.

Postoperatively, all patients received standard antibiotic prophylaxis and wore a rigid cervical collar for 4 to 6 weeks. Routine follow-up was conducted at predefined intervals to assess fusion status and clinical recovery.

### 2.3. Clinical and radiographic evaluation

Clinical outcomes were assessed preoperatively and at 6 and 24 months postoperatively using the JOA score for cervical myelopathy, the VAS for neck pain, and the NDI. The JOA recovery rate was calculated using the Hirabayashi formula: recovery rate (%) = (postoperative JOA − preoperative JOA)/(17 − preoperative JOA) × 100.

Radiological assessments included lateral cervical spine radiographs and CT scans obtained at the same follow-up time points. Sagittal alignment was evaluated using the C2 to C7 Cobb angle, defined as the angle between the inferior endplate of C2 and the inferior endplate of C7 on lateral radiographs. A positive value indicated cervical lordosis.

Implant subsidence was defined as a decrease in the anterior or posterior height of the fused segment of ≥ 3 mm compared to the immediate postoperative measurement. Measurements were performed using calibrated lateral radiographs, and the greater of the 2 height losses (anterior vs posterior) was recorded.

Interbody fusion was determined based on the presence of continuous trabecular bone bridging between the graft and adjacent endplates and the absence of motion or radiolucent gaps across the fusion segment. Fusion status was confirmed by dynamic radiographs and, when necessary, by CT scan. Two independent spine surgeons blinded to group allocation evaluated all radiographs, and discrepancies were resolved by consensus.

### 2.4. Statistical analysis

All statistical analyses were performed using R software (version 4.3.0; R Foundation for Statistical Computing, Vienna, Austria). The Shapiro–Wilk test was used to assess the normality of continuous variables. Normally distributed variables were expressed as mean ± standard deviation and compared using the independent samples *t* test or paired *t* test, as appropriate. Non-normally distributed data were presented as median and interquartile range and analyzed using the Mann–Whitney *U* test or Wilcoxon signed-rank test. Categorical variables were presented as counts and percentages and compared using the chi-square test or Fisher exact test, as appropriate.

To reduce selection bias and confounding, propensity scores were estimated using multivariate logistic regression based on baseline covariates. A 1:1 nearest-neighbor matching without replacement was conducted using a caliper width of 0.2. Matching variables included age, sex, comorbidities (hypertension, diabetes, smoking status, alcohol use), BMD, preoperative functional scores (JOA, VAS, and NDI), and radiological parameters (C2–C7 Cobb angle and spinal canal occupation ratio).

A 2-tailed *P*-value of < .05 was considered statistically significant.

## 3. Results

### 3.1. Patient demographics and baseline characteristics

Among the 133 patients who met the inclusion criteria, 41 (30.8%; follow-up rate: 80.4%, 41 of 51 eligible patients) were included in the NEAVBF group, and 92 (69.2%; follow-up rate: 81.4%, 92 of 113 eligible patients) were included in the TCF group before propensity score matching. Significant differences were observed between the 2 groups in terms of BMD (T-score: −2.86 ± 0.27 vs −2.72 ± 0.26, *P* = .006) and smoking status (41.5% vs 21.7%, *P* = .033). No significant differences were found in other demographic, radiographic, or clinical variables, including age, sex, comorbidities, spinal canal occupying ratio, Cobb angle, or preoperative patient-reported outcome measures (VAS, NDI, and JOA scores) (Table 1).

After propensity score matching, 82 patients were included in the NEAVBF (n = 41) and TCF (n = 41) groups. No significant differences were observed between the 2 groups in terms of baseline demographics, BMD, comorbidities, radiographic parameters, or preoperative functional scores (Table 1), indicating adequate baseline balance after matching.

### 3.2. Operative factors and complications

There were no significant differences between the NEAVBF and TCF groups in terms of operative time (160.06 ± 18.20 vs 160.12 ± 20.85 minutes, *P* = .988) or intraoperative blood loss (248.30 ± 54.38 vs 256.24 ± 56.10 mL, *P* = .517) (Table 2).

**Table 2:** Operative factors and complications.

	NEAVBF (n = 41)	TCF (n = 41)	*P*-value
Operative factors			
Operative time	160.06 ± 18.20	160.12 ± 20.85	.988
Blood loss	248.30 ± 54.38	256.24 ± 56.10	.517
Complications			
Superficial surgical site infection	1 (2.4%)	0 (0.0%)	1.000
Postoperative dysphagia	0 (0.0%)	1 (2.4%)	1.000

NEAVBF = novel expandable artificial vertebral body fusion, TCF = titanium cage fusion.

Regarding perioperative complications, 1 patient (2.4%) in the NEAVBF group developed a superficial surgical site infection, whereas 1 patient (2.4%) in the TCF group experienced postoperative dysphagia. No significant difference was observed in the incidence of complications between the 2 groups (*P* = 1.000 for both).

### 3.3. Radiographic outcomes

Both groups demonstrated significant improvements in C2 to C7 cervical lordosis following surgery. In the NEAVBF group, the mean Cobb angle increased from 8.14 ± 2.08° preoperatively to 10.56 ± 2.17° at 6 months (*P* < .001) and further to 11.16 ± 2.05° at 2 years postoperatively (*P* < .001). Similarly, in the TCF group, lordosis improved from 8.48 ± 2.22° to 9.53 ± 2.26° at 6 months (*P* < .001) and 9.76 ± 2.25° at 2 years (*P* < .001). However, the improvement was more pronounced in the NEAVBF group, which exhibited significantly greater C2 to C7 lordosis than the TCF group at both postoperative time points (6 months: *P* = .038; 2 years: *P *= .004) (Table 3).

**Table 3:** Radiographic findings.

		NEAVBF	TCF	*P*-value
C2–C7 lordosis	Preop	8.14 ± 2.08	8.48 ± 2.22	.480
	Postop 6 mo	10.56 ± 2.17†	9.53 ± 2.26†	.038*
	Postop 2 yr	11.16 ± 2.05†,‡	9.76 ± 2.25†,‡	.004*
Implant subsidence rate, n (%)	Postop 6 mo	5 (12.2%)	13 (31.7%)	.060
	Postop 2 yr	11 (26.8%)	22 (53.7%)	.024*
Fusion rate, n (%)	Postop 6 mo	29 (70.7%)	21 (51.2%)	.112
	Postop 2 yr	40 (97.6%)‡	38 (92.7%)‡	.616

NEAVBF = novel expandable artificial vertebral body fusion, TCF = titanium cage fusion.

* Statistically significant difference between the 2 groups.

† Statistically significant change compared with the preoperative measurement.

‡ Statistically significant change compared with the postoperative 6 months measurement.

With respect to implant subsidence, the NEAVBF group showed a lower incidence at both 6 months (12.2% vs 31.7%) and 2 years (26.8% vs 53.7%) compared to the TCF group. Although the difference at 6 months did not reach statistical significance (*P* = .060), it became significant at the 2-year follow-up (*P* = .024), indicating better long-term implant stability in the NEAVBF group.

Fusion rates increased over time in both groups. At 6 months, the fusion rate in the NEAVBF group was higher than that in the TCF group (70.7% vs 51.2%), although this difference did not reach statistical significance (*P* = .112). By 2 years, both groups achieved high fusion rates (97.6% vs 92.7%), with no significant intergroup difference (*P* = .616). Notably, the rate of fusion progression was steeper in the NEAVBF group, suggesting a more consistent early fusion trend.

### 3.4. Patient-reported outcome measures

Both groups exhibited significant improvements in patient-reported outcomes after surgery. VAS scores decreased markedly in both the NEAVBF and TCF groups at 6 months compared with baseline (NEAVBF: 5.27 ± 1.05 to 1.95 ± 0.30; TCF: 5.46 ± 1.07 to 2.46 ± 0.29; *P* < .001 for both). Notably, the NEAVBF group reported significantly lower VAS scores than the TCF group at 6 months (*P* < .001), while no significant difference was found at 2 years (*P* = .913), indicating comparable long-term pain control (Table 4).

**Table 4:** Patient-reported outcome measures.

		NEAVBF	TCF	*P*-value
VAS	Preop	5.27 ± 1.05	5.46 ± 1.07	.408
	Postop 6 mo	1.95 ± 0.30†	2.46 ± 0.29†	<.001*
	Postop 2 yr	2.00 ± 0.31†	1.99 ± 0.30†,‡	.913
NDI	Preop	31.85 ± 8.77	31.29 ± 8.29	.767
	Postop 6 mo	18.84 ± 4.11†	17.12 ± 4.23†	.065
	Postop 2 yr	13.51 ± 3.41†,‡	14.07 ± 3.93†,‡	.494
JOA score	Preop	10.85 ± 0.34	10.97 ± 0.32	.102
	Postop 6 mo	14.42 ± 0.52†	14.13 ± 0.54†	.013*
	Postop 2 yr	14.98 ± 0.53†,‡	15.10 ± 0.47†,‡	.287
JOA recovery rate	Postop 6 mo	58.34 ± 6.83	52.53 ± 7.86	<.001*
	Postop 2 yr	67.42 ± 7.61‡	68.69 ± 7.04‡	.436

JOA = Japanese Orthopedic Association, NDI = Neck Disability Index, NEAVBF = novel expandable artificial vertebral body fusion, TCF = titanium cage fusion, VAS = Visual Analogue Scale.

* Statistically significant difference between the 2 groups.

† Statistically significant change compared with the preoperative measurement.

‡ Statistically significant change compared with the postoperative 6 months measurement.

NDI scores also improved over time in both groups. Although no significant intergroup difference was observed at either follow-up point (6 months: *P* = .065; 2 years: *P* = .494), both groups demonstrated significant reductions from preoperative values (*P* < .001), with further improvement noted between 6 months and 2 years (*P* < .001).

JOA scores increased significantly in both groups at 6 months postoperatively compared with baseline (*P* < .001), with the NEAVBF group achieving a significantly higher JOA score than the TCF group (14.42 ± 0.52 vs 14.13 ± 0.54, *P* = .013). However, at the 2-year follow-up, this difference was no longer significant (*P* = .287), and both groups maintained stable functional improvement (*P* < .001 for both).

Regarding the JOA recovery rate, the NEAVBF group demonstrated a significantly higher rate at 6 months than the TCF group (58.34% ± 6.63 vs 52.53% ± 7.86, *P* < .001). However, by 2 years, both groups achieved comparable recovery rates (67.42% ± 7.61 vs 68.69% ± 7.04, *P* = .436), suggesting that the initial advantage of NEAVBF may diminish over time.

## 4. Discussion

In this retrospective propensity score-matched study, we evaluated the clinical and radiographic outcomes of ACCF using either NEAVBF or TCF in patients with cervical OPLL complicated by osteoporosis. The primary findings demonstrated that although both surgical techniques resulted in significant and sustained improvements in pain, neurological function, and sagittal alignment, the NEAVBF group exhibited superior early postoperative outcomes, including significantly greater restoration of cervical lordosis, lower implant subsidence rates at 6 months, and higher neurological recovery rates.

Importantly, the incidence of implant subsidence remained significantly lower in the NEAVBF group even at 2 years postoperatively (26.8% vs 53.7%, *P* = .024), suggesting superior long-term biomechanical stability. Although fusion rates, pain relief, and functional recovery were comparable between the 2 groups at final follow-up, the early and sustained advantages observed in the NEAVBF group may have important clinical implications for promoting spinal stability and reducing the risk of implant-related complications in osteoporotic patients. These findings support the potential benefits of expandable vertebral body systems in high-risk cervical populations, where optimized load sharing and endplate preservation are critical.

Several previous studies have investigated the outcomes of ACCF using titanium cages or expandable vertebral body systems in patients with cervical degenerative disease or OPLL.^[14–16]^ Titanium mesh cages are widely used due to their structural simplicity and affordability; however, their use has been associated with relatively high rates of implant subsidence, particularly in osteoporotic patients.^[17]^ Reported subsidence rates following ACCF with titanium cages range from 20% to over 50%, depending on patient bone quality and surgical technique.^[8,18,19]^ Similarly, fusion rates using titanium cages typically exceed 90% at long-term follow-up, although early segmental stability may be compromised in individuals with low BMD.^[20]^

Expandable vertebral body systems, such as the NEAVBF device evaluated in this study, have been proposed as viable alternatives that offer intraoperative height adjustment and improved endplate contact.^[21,22]^ Prior biomechanical studies have demonstrated that expandable cages achieve better load distribution and reduce peak stress at the endplate interface, thereby lowering the risk of subsidence in osteoporotic models.^[23,24]^ However, clinical evidence comparing expandable and titanium cages in high-risk populations remains limited.

To our knowledge, this is one of the first studies to directly compare NEAVBF and TCF in patients with cervical OPLL complicated by osteoporosis using propensity score matching. Our findings reinforce the theoretical biomechanical advantages of NEAVBF systems, particularly in reducing early and long-term implant subsidence without compromising fusion outcomes. Furthermore, the ability to restore cervical alignment more effectively with an expandable device may contribute to improved neurological recovery during the early postoperative phase.

The early postoperative benefits observed in the NEAVBF group may be largely attributed to the biomechanical properties of the expandable cage design.^[25]^ Unlike conventional titanium mesh cages, the NEAVBF device permits intraoperative expansion and precise adjustment of implant height and lordotic angle, allowing for optimal restoration of cervical sagittal alignment and improved endplate contact.^[26]^ Enhanced conformity between the cage and endplates may facilitate more uniform axial load transmission, thereby minimizing point-loading and reducing the likelihood of implant subsidence: an effect particularly critical in osteoporotic bone with compromised endplate integrity.^[23]^

In addition, by better restoring anterior column height and sagittal balance, the NEAVBF system may reduce micromotion at the fusion site during early healing, promoting early bony integration. This mechanical stability could explain the higher early fusion rates observed in the NEAVBF group. However, as biological fusion progresses over time, the initial differences between groups may diminish, resulting in similar long-term outcomes at 2 years. Given the rising prevalence of osteoporosis in aging populations and the unique biomechanical challenges it poses to spinal reconstruction, expandable vertebral body systems may offer distinct clinical advantages.^[27]^ Their ability to optimize fit, alignment, and load distribution makes them particularly well suited for patients with poor bone quality.^[28]^

Several limitations of this study should be acknowledged. First, the retrospective nature of the study introduces inherent risks of selection bias and limits causal inference. Although propensity score matching was used to control for baseline differences, residual confounding cannot be entirely excluded.^[29]^ Second, the sample size was relatively small, especially after matching, which may limit the statistical power to detect subtle differences, particularly regarding rare complications.^[30]^ Third, the study was conducted at a single institution by a single surgical team, which, while ensuring consistency in technique, may limit the generalizability of the findings. Fourth, certain radiographic assessments (such as fusion status and subsidence) relied in part on subjective interpretation, despite being evaluated using standardized criteria by independent reviewers.^[31]^ Lastly, the absence of external validation or multi-center data restricts the broader applicability of our results.

Future research is warranted to validate these findings in larger, prospective, multi-center randomized controlled trials. While our current study represents the largest feasible single-center cohort within the defined period of early NEAVBF adoption, we acknowledge the limitations inherent in its retrospective design and relatively small sample size. Expanding the study duration retrospectively would not substantially increase the number of eligible cases due to the recent introduction of the NEAVBF system at our institution. Therefore, collaboration across multiple centers will be essential to achieve larger cohorts and ensure adequate statistical power. Such prospective studies will provide higher-level evidence and enhance the generalizability of outcomes across diverse populations and surgical practices. In addition, longer follow-up periods beyond 5 years are needed to assess the durability of reconstruction, long-term implant stability, and the incidence of delayed complications such as adjacent segment disease or implant failure. Further investigations should also explore the use of expandable vertebral body systems in more complex scenarios, such as multilevel corpectomies or patients with more advanced osteoporosis. Comparative studies evaluating cost-effectiveness, reoperation rates, and patient-reported outcomes will be critical to determine the broader clinical value of these devices. Given the increasing burden of osteoporosis in aging societies, expandable vertebral body systems may represent a promising strategy to enhance early spinal stability and improve recovery in high-risk patients undergoing anterior cervical reconstruction. Moreover, our study has additional limitations to acknowledge. Another limitation is that we did not include data on systemic inflammatory markers, as these were not routinely collected in our standard perioperative protocol. While this study focused primarily on clinical outcomes and radiographic parameters, future prospective research should consider incorporating inflammatory biomarkers to investigate their potential association with fusion rates, implant stability, and healing processes in osteoporotic patients.

In summary, this study demonstrates that the use of a novel expandable artificial vertebral body system may offer biomechanical and clinical advantages over traditional titanium cages in patients with cervical OPLL and osteoporosis undergoing ACCF. The observed reduction in implant subsidence and improved early neurological recovery highlight its potential role in optimizing surgical outcomes in this high-risk population. While long-term functional results were comparable between groups, the early postoperative benefits suggest that expandable systems may be particularly valuable for enhancing stability during the critical initial healing phase.

## Acknowledgments

The authors thank the editors and reviewers for their valuable comments and suggestions on the manuscript.

## Author contributions

**Conceptualization:** Yaqi Li, Hanhui Liu.

**Data curation:** Yaqi Li, Kexi Yang, Jialing Liang.

**Formal analysis:** Yaqi Li, Kexi Yang.

**Investigation:** Yaqi Li, Kexi Yang, Zhancheng Liang, Jialing Liang, Hanhui Liu.

**Methodology:** Yaqi Li, Hanhui Liu.

**Project administration:** Hanhui Liu.

**Supervision:** Hanhui Liu.

**Validation:** Zhancheng Liang, Jialing Liang, Hanhui Liu.

**Visualization:** Kexi Yang.

**Writing – original draft:** Yaqi Li.

**Writing – review & editing:** Yaqi Li, Kexi Yang, Zhancheng Liang, Jialing Liang, Hanhui Liu.
